# A New Pattern of Brain and Cord Gadolinium Enhancement in Molybdenum Cofactor Deficiency: A Case Report

**DOI:** 10.3390/children10061072

**Published:** 2023-06-17

**Authors:** Giulia Lucignani, Leonardo Vattermoli, Maria Camilla Rossi-Espagnet, Alessia Guarnera, Antonio Napolitano, Lorenzo Figà-Talamanca, Francesca Campi, Sara Ronci, Carlo Dionisi Vici, Diego Martinelli, Carlo Gandolfo, Daniela Longo

**Affiliations:** 1 Functional and Interventional Neuroradiology Unit, Bambino Gesù Children’s Hospital IRCCS, Piazza Sant’Onofrio, 4, 00165 Rome, Italy; 2 Department of Diagnostic Imaging and Interventional Radiology, Tor Vergata University of Rome, 00133 Rome, Italy; 3 Neuroradiology Unit, NESMOS Department Sant’Andrea Hospital, La Sapienza University, Via di Grottarossa, 1035-1039, 00189 Rome, Italy; 4 Medical Physics Unit, Bambino Gesù Children’s Hospital IRCCS, Piazza Sant’Onofrio, 4, 00165 Rome, Italy; 5 Neonatal Intensive Care Unit, Bambino Gesù Children’s Hospital IRCCS, Piazza Sant’Onofrio, 4, 00165 Rome, Italy; 6 Department of Pediatric Specialties and Liver-Kidney Transplantation, Division of Metabolic Diseases and Drug Biology, Bambino Gesù Children’s Hospital IRCCS, Piazza Sant’Onofrio, 4, 00165 Rome, Italy; 7 Unit of Metabolism, Bambino Gesù Children’s Hospital IRCCS, Piazza Sant’Onofrio, 4, 00165 Rome, Italy

**Keywords:** molybdenum cofactor deficiency, inborn error metabolism, MRI, metabolic

## Abstract

Molybdenum cofactor deficiency (MoCD) is a rare and severe autosomal recessive in-born error of metabolism caused by the mutation in MOCS1, MOCS2, MOCS3 or GEPH genes, with an incidence ranging between 1 in 100,000 and 200,000 live births. The clinical presentation with seizures, lethargy and neurologic deficits reflects the neurotoxicity mediated via sulphite accumulation, and it occurs within the first hours or days after birth, often leading to severe neurodegeneration and the patient’s death within days or months. The Imaging of Choice is a brain-specific MRI technique, which is usually performed without contrast and shows typical radiological findings in the early phase, such as diffuse cerebral oedema and infarction affecting the cortex and the basal ganglia and the white matter, as well as in the late phase, such as multicystic encephalomalacia. Our case report represents a novelty in the field, since the patient underwent a contrast-enhanced MRI to exclude a concomitant infectious disease. In the frame of the clinical presentation and laboratory data, we describe the MoCD Imaging findings for MRI morphological and advanced sequences, presenting a new contrast-enhanced MRI pattern characterized by the diffuse and linear leptomeningeal enhancement of brain, cord and spinal roots. The early identification of molybdenum cofactor deficiency is crucial because it may lead to the best multidisciplinary therapy for the patient, which is focused on the prompt and optimal management of the complications.

## 1. Introduction

Molybdenum cofactor deficiency (MoCD) is a rare and challenging autosomal recessive in-born error of metabolism with an incidence ranging between 1 in 100,000 and 200,000 live births [1], though fewer than 150 cases have been described so far [2]. It is caused by the mutation in MOCS1 (Molybdenum Cofactor Synthesis 1), MOCS2 (molybdenum cofactor synthesis 2), MOCS3 (molybdenum cofactor synthesis 3) and GPHN (Gephyrin) genes, which determine the deficit of the molybdenum cofactor-dependent enzymes sulphite oxidase, xanthine dehydrogenase and aldehyde oxidase, resulting in sulphite accumulation [2,3,4].

Sulphites are particularly toxic for neurons and white matter [5]. Therefore, the clinical manifestations of the disease usually occur during the first hours or days after birth, with severe seizures, feeding difficulties, lethargy, and neurologic deficit, including motor palsy [1,2,3,4,6]. Other disease features are microcephaly and ophthalmological alterations, such as lens dislocation, spherophakia and nystagmus [1]. Severe neurodegeneration occurs in a few days or months, often leading to the patient’s death [1,3,7].

Patients usually undergo MRI without contrast administration and present typical findings, such as diffuse cerebral oedema and infarction affecting the cortex and the basal ganglia and the white matter, followed by multicystic encephalomalacia [1,8].

Our case report represents a novelty in the field, since the patient underwent a contrast-enhanced MRI to exclude a concomitant infectious disease. In the frame of the clinical presentation, we describe the MoCD Imaging features identifiable in the morphological and advanced sequences, such as MRI spectroscopy, with a particular focus on the newly described Imaging findings related to the contrast administration, which are characterized by the diffuse and linear leptomeningeal enhancement of brain, cord and spinal roots. The early identification of a rare and challenging pathology, such as molybdenum cofactor deficiency, may lead to the best multidisciplinary therapy for the patient, which is focused on the prompt and optimal management of the complications.

## 2. Case Report

A female newborn of 36 h of life, who was the second child of second-degree related parents and born at 39 weeks of gestational age via eutocic delivery, was admitted to our institution for generalized seizures. The admission blood test demonstrated metabolic acidosis (pH 7.33, HCO_3_^−^ 11.8 mmol/L) and increased inflammation parameters (CRP 15.7 mg/L).

No complications were reported during the pregnancy or at birth. The patient did not present any dysmorphic features. TORCH screening, as well as vaginal and rectal swabs, were negative. Family history reported the death of the maternal aunt due to epilepsy at 25 years of age and that of a first-degree cousin for cerebral haemorrhage at 9 years of age.

The patient was transferred to the neonatal intensive care unit of our institution and received the following therapy: seizures were treated with phenobarbital, phenytoin and midazolam; treatment of hyperammonemia and hyperlacticaemia was started with progressive normalization of the values; and antibiotic therapy was administered due to suspicion of infection.

EEG (electroencephalogram) was performed and showed global slowing, unstructured and hypovolted brain activity with subsequent multifocal electrographic crises. Lacosamide therapy was immediately started, providing limited benefit.

**Figure 1:**
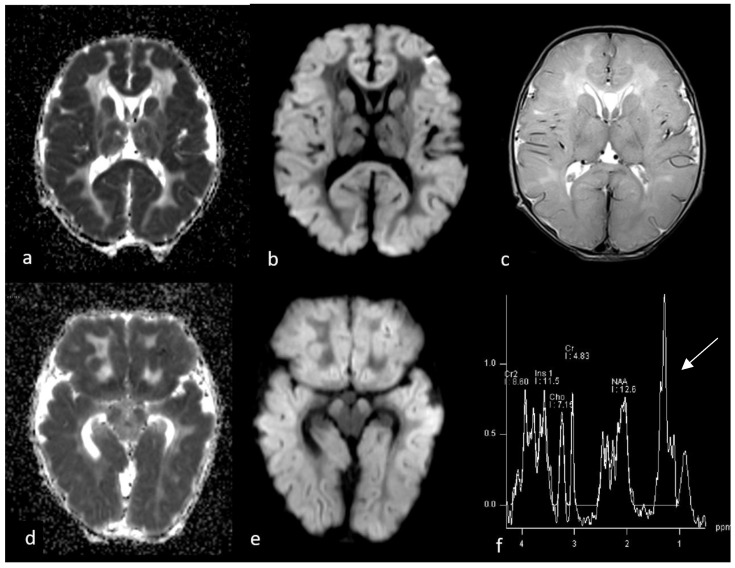
First MRI performed at 3 days after birth. Axial ADC (**a**,**d**), DWI (**b**,**e**) and T2WI sequences (**c**) show diffuse brain swelling associated with diffusion restriction involving cortical grey matter, subcortical which matter, basal ganglia and cerebral peduncles, with relative sparing of deep white matter. Single-voxel MR spectroscopy graph (**f**) was performed by putting a voxel in basal ganglia and showed an increase in lactate/lipid peak (arrow) and a decrease in NAA.

**Figure 2:**
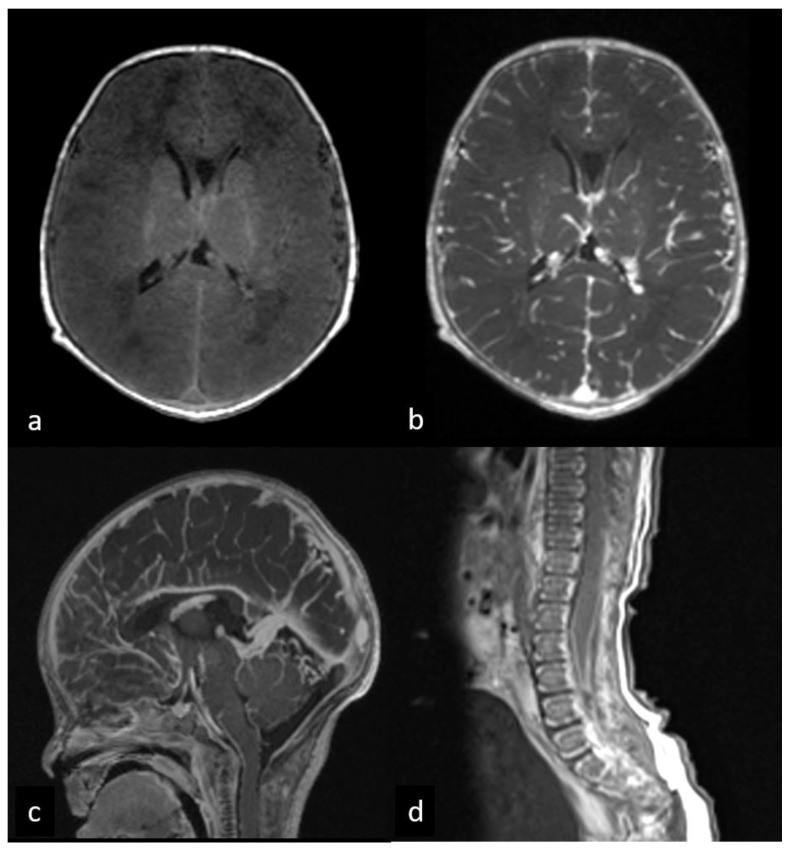
First MRI performed at 3 days after birth before and after gadolinium administration. Pre- (**a**) and post-contrast (**b**,**c**) brain axial T1WI shows a diffuse cerebral leptomeningeal enhancement. Post-contrast sagittal T1WI of lumbar spine (**d**) demonstrates leptomeningeal enhancement along the spinal cord and cauda equina roots.

Following a relapse in seizures, a contrast-enhanced brain and spine MRI was performed at three days after birth (Figure 1 and Figure 2). MRI was performed using a 3T scanner (Magnetom Skyra, Siemens, Erlangen, Germany) with the protocol outlined in Table 1. Moreover, a single-voxel MRS (magnetic resonance spectroscopy) was acquired through positioning the VOI (volume of interest) in the basal ganglia and capsular regions of the left hemisphere.

MRI showed diffuse cytotoxic brain edema characterized by T2 hyperintensity and T1 hypointensity, as well as intense DWI/ADC signal restriction, which symmetrically and diffusively extended to the cerebral cortex, the basal ganglia and thalami, with coexisting bilateral huncal herniation. Cerebellum, dorsal pons and medulla oblongata were spared, and there was no contralateral brain shift. The ventricular system seemed compressed, and subarachnoid spaces were obliterated. Single-voxel MRS (Magnetic Resonance Spectroscopy) identified low NAA (N-Acetyl-Aspartate) and a high lactate/lipid peak in the basal ganglia. After gadolinium administration, diffuse and linear leptomeningeal enhancement of brain, spinal cord, and cauda equina roots were detected (Figure 1 and Figure 2).

Lumbar puncture, sepsis workup and extensive neonatal screening were performed, though no positive result was identified.

Additional metabolic blood and urinary tests demonstrated low levels of uric acid (<0.2 mg/dL vs. nv: 2.4–5.7 mg/dL) in the blood, as well as high urinary levels of sulphites (8.21 mcM/L vs. nv: 50–3.50 mcM/L) and xanthine (442 M/mM creat vs. nv: 1–65 M/mM creat).

Clinical, laboratory and Imaging findings were suggestive of Molybdenum cofactor deficiency, which was subsequently confirmed via MOCS2 gene mutation through genetic analysis performed using the peripheral blood of the proband. In particular, the analysis of amplified segments of coding regions and exon–intron junctions (±5 bp) of genes related to the clinical indication detected the presence of the homozygous mutation of c.377 + 1 G > A in the canonical splicing site of the MOCS2 gene (rs1389817466). The variant, which was also segregated from the peripheral blood of both heterozygous carrier parents, can be classified, according to the ACMG SF v3.1 guidelines, as pathogenic (class 5) [9].

**Figure 3:**
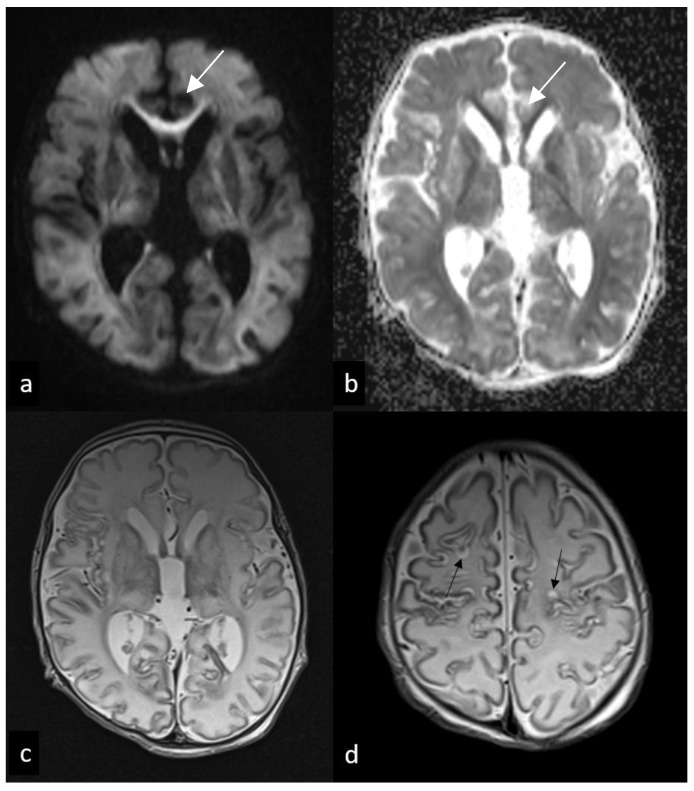
Follow-up MRI performed 7 and 12 days after birth. Axial DWI (**a**), ADC, (**b**) and T2WI (**c**,**d**) show mild diffusion restriction involving subcortical white matter and corpus callosum ((**a**,**b**), arrows), suggesting an acute wallerian degeneration, as well as diffuse ventricular enlargement (**c**) and subcortical cavitations (black arrows in (**d**)).

**Figure 4:**
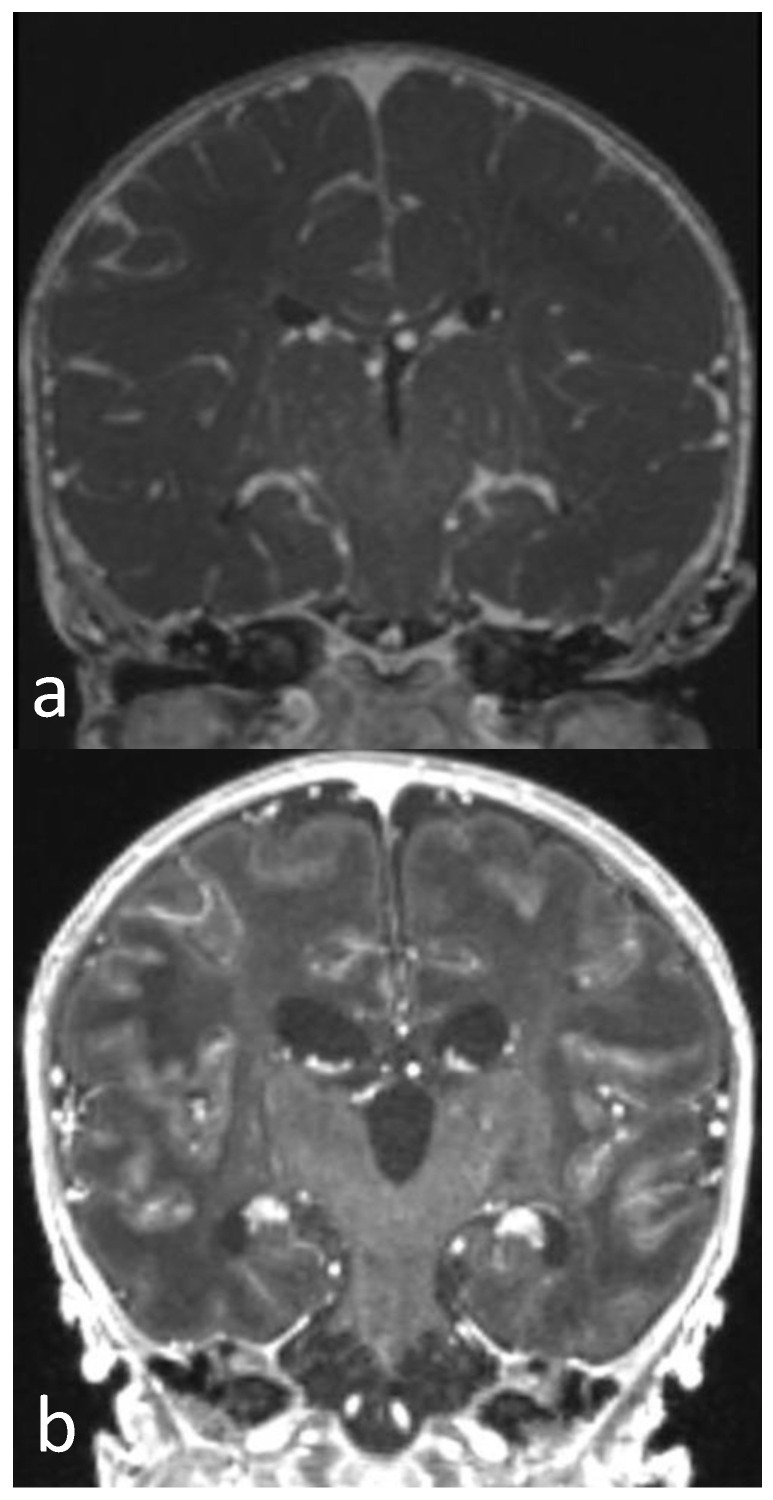
Post-contrast T1WI sequence performed 3 (**a**) and 12 days after birth (**b**). Diffuse and linear leptomeningeal enhancement in first MRI (**a**) persists in follow-up MRI (**b**). Diffuse brain swelling (**a**) evident in first MRI is markedly reduced in second MRI (**b**), in which a reduction in brain oedema and dilatation of ventricular system and subarachnoid spaces are shown.

Follow-up MRIs were performed 9 and 12 days after birth, demonstrating a global reduction in cytotoxic oedema, which persisted in the basal ganglia, internal capsule, cerebral peduncles, and corpus callosum, being probably related to Wallerian degeneration. Concomitant ventricular and subarachnoid space expansion was consistently identified. Curvilinear hyperintensity on T1WI appears in the frontal and parietal cortex, suggesting the presence of cortical laminar necrosis, which coexisted with small cortical cystic presenting low T1 signal and T2 hyperintensity. Single-voxel MRS (Magnetic Resonance Spectroscopy) confirmed a low NAA (N-Acetyl-Aspartate) and a high lactate/lipid ratio in the basal ganglia. Contrast-enhanced T1 showed the persistence of leptomeningeal enhancement in the brain, spinal cord and cauda equina roots (Figure 3 and Figure 4).

The follow-up EEG demonstrated burst-suppression activity, with phases of suppression of electrical activity lasting up to 30 s and recurrent electrical crises lasting up to 25 s in the central–temporal regions of the brain, with a peculiar alternation between the hemispheres.

At 19 days after birth, the patient presented a rapid decline in vital functions characterized by a sudden and severe bradycardia and desaturation, which led to the patient’s death.

## 3. Discussion

Our case report represents a novelty since it, firstly, describes a new pattern of MRI brain and cord gadolinium enhancement in Molibdenum cofactor deficiency, i.e., diffuse and linear brain, cord and spinal root enhancement at 3 days after birth, which persisted with similar characteristics at 12 days after birth.

MoCD is a rare and severe autosomal recessive in-born error of metabolism, the incidence of which ranges between 1 in 100,000 and 200,000 live births; it involves a challenging diagnosis since the clinical presentation is not specific and may mimic severe pathologies characterized by a similar clinical presentation, such as perinatal hypoxic-ischemic encephalopathy (HIE) [1,2,3,6,8].

MoCD leads to deficiencies in sulphite oxidase, xanthine dehydrogenase, the mitochondrial amidoxime-reducing component and aldehyde oxidase, which are the four human molybdoenzymes. A deficit of these enzymes causes sulphite accumulation and carries dire consequences, leading to the patient’s death [10].

There are several mechanisms through which sulphites may cause cell damage. Sulphites can cause cellular damage by reacting with lipids and proteins and forming radicals that damage nucleic acids and attack disulfide bonds. Sulfites can damage the mitochondrial membrane or interfere with the tricarboxylic acid cycle, causing ATP loss and magnesium release. Finally, the excessive production of amino acids containing sulfur (e.g., S-sulfocyststeine) may cause excessive activation of the NMDA receptor, leading to increased values of intracellular calcium and magnesium, thus resulting in excitotoxic neuronal damage. Toxic concentrations of magnesium may exacerbate NMDA-induced excitotoxic damage [5].

Among the various subgroups of MoCD, which are characterized by the mutation in MOCS1, MOCS2, MOCS3 or GEPH genes, the mutation in MOCD2 follows an autosomal recessive pattern of inheritance and is extremely rare, and patients may show two different forms: the early-onset (beginning during the neonatal period) and late-onset forms. According to the literature, only 11 cases of early-onset patients were previously reported [11].

In the early-onset form of MoCD, symptoms starting in the first days of life encompass severe encephalopathy, including refractory seizures, hypotonia, apnea and feeding difficulties. Most patients present with facial dysmorphism, i.e., a prominent forehead, narrow bifrontal diameter, deep-set eyes, elongated palpebral fissures, plump cheeks, small nose, long philtrum, and thick lips. Patients with late-onset MoCD typically present with milder symptoms. During an acute episode, such as an infection, it is possible to have neurologic decompensation with alteration of mental status, nystagmus and dystonia, as well as changes in tone and motility [12].

Antenatal diagnosis is rarely performed, and routes are used in the measurement of the enzyme activity in chorionic villus samples or sulfocysteine levels in amniotic fluid. DNA analysis may also confirm the diagnosis [10].

MRI is the imaging modality of choice that shows two main phases.

In the early presentation, brain MRI demonstrates diffuse oedema within the cerebral hemispheres, basal ganglia and thalami, with more acute, focal and symmetrical changes within the globus pallidi, cerebral peduncles of the midbrain and subthalamic regions. Focal changes included swelling, with a hyperintense signal observed on T2/FLAIR and diffusion-weighted imaging, while a hypointense signal is observed when calculating apparent diffusion coefficient maps with restricted diffusion. This patter is very similar to HIE; however, according to the literature, curvilinear areas of reduced signal intensity may appear at the grey/white matter junction after initial oedema subsides, suggesting the presence of haemorrhagic deposits and laminar necrosis that are also more specific to MoCD. The caudate and thalami are rarely affected, though and is always affected in MoCD. MRS detects an increase in lactates and a decrease in NAA, probably due to the sulphites’ toxicity in mitochondria [2,3,5,6,7,13]. These MRS findings are like the ones occurring in perinatal hypoxic–ischemic encephalopathy, as well as the differential diagnosis roots in the normal or elevated choline levels in MoCod with respect to HIE [1]. In the late stage, MRI reveals extensive subcortical and periventricular white matter loss, cystic encephalomalacia and hyperintensity in white matter, dysgenesis of the corpus callosum and ventriculomegaly with generalized volume loss. In addition, focal polymicrogyria and focal agyria in the cerebral hemisphere at the cortical location are described. [1,2,3,4,6,7,8]. Patients usually undergo MRI without contrast; therefore, no patterns of MRI enhancement were previously described in MoCd.

Clinical presentation, laboratory tests and imaging suggest MoCd, which requires genetic confirmation for diagnosis [1,4,7]. The identification of biallelic pathogenic variants in GPHN, MOCS1, MOCS2 or MOCS3 allows a definitive diagnosis of molybdenum cofactor deficiency. If genetic testing is unavailable, it is possible to study the activity of sulphite oxidase enzyme in cultured fibroblasts, which is significantly reduced [12].

MRIs performed at 3 and 12 days after birth typically show the two-phase radiological presentation characterized, respectively, by the diffuse cerebral cytotoxic oedema referenced in the literature [13], and a very rapid evolution to a subsequent multicystic encephalomalacia, with subcortical cysts. This aspect is rarely found in patients with HIE, which could support the metabolic hypothesis [14]. The crucial novelty of our case is the description of a new MRI gadolinium enhancement, which is characterized by linear and diffuse leptomeningeal enhancement evident in the brain, cord and spinal roots, and which persisted in the follow-up MRI.

Our hypothesis is that the leptomeningeal enhancement could be related to the alteration and increased permeability of the blood–brain barrier caused by mitochondrial and endothelial damage because of toxic sulphite accumulation, causing severe perivascular inflammation and infiltration in response to tissue hypoxia [15,16]. This pathogenetic mechanism was previously described in other central nervous system autoimmune inflammatory diseases and some metabolic diseases, such as hepatic encephalopathy, lysosomal disorders and mitochondrial diseases, such as POLG-related disease [17,18,19,20].

Genetic counselling confirmed the suspicion of MoCd, since we performed the clinical exome sequencing test on the new-born child and her parents. A homozygous mutation c.377 + 1 G > A was detected in a canonical splice site of the MOCS2 gene (rs1389817466). The variant, which was segregated from both heterozygous carrier parents, is described in the scientific literature as being in association with autosomal recessive molybdenum deficiency [9].

Although we provided our patient with the best supportive therapy available and started the treatment with cPMP, at only 19 days after birth, our patient presented a rapid decline in vital functions characterized by a sudden and severe bradycardia and desaturation, which led to the patient’s death.

The disorder is associated with a poor prognosis, with irreversible neurological sequelae, and may lead to death in the first weeks of life or, in rare cases, after a few months of life [1,3,7]. Early and multidisciplinary therapy is mostly focused on the prompt and optimal management of disease complications, since there is no cure for MoCd. Some studies described early MoCd treatment with cPMP, which should be administered before the onset of cystic degeneration to alleviate the patient’s clinical condition without correcting the biochemical defect [1,3,8]. This therapy is extremely effective in reducing sulfite toxicity and rebalancing biochemical homeostasis; unfortunately, the clinical outcome depends on the severity of the cerebral lesion identified at the beginning of treatment [21].

## 4. Conclusions

Our case report represents a description of a new and peculiar MRI gadolinium-enhanced pattern in Molibdenum cofactor deficiency. In particular, we observed linear and diffuse leptomeningeal enhancement in the brain, cord and spinal roots. The significance of this finding is critical, since it may trigger the clinical suspicion of MoCD deficiency and encourage the performance of the clinical and genetic tests needed to reach a final diagnosis.

## Figures and Tables

**Table 1:** MRI brain and spine protocol.

	Brain				Spine	
**Sequences**	**3D T1 MPRAGE**	**SE T2WI**	**DWI/ADC**	**MRS**	**T1**	**T2**
TR (ms)	2300	10,320	8100	1980	1890	4000
TE (ms)	3.4	122	76	30	30	122
Field of view	256	180	200		280	280
Slice thickness (mm)	1	2	2		2	2

## Data Availability

Not applicable.

## Abbreviations

MoCD	molybdenum cofactor deficiency
MOCS1	molybdenum cofactor synthesis 1
MOCS2	molybdenum cofactor synthesis 2
MOCS3	molybdenum cofactor synthesis 3
GPHN	gephyrin
MRI	magnetic resonance imaging
WI	weighted image
ST	slice thickness
TR	repetition time
TE	echo time
FOV	field of view
pH	potential of hydrogen
HCO^3^	bicarbonate
CRP	c-reactive protein
DWI	diffusion-weighted image
ADC	apparent diffusion coefficient
MRS	magnetic resonance spectroscopy
NAA	N-acetyl aspartate
HIE	hypoxic–ischemic encephalopathy
cPMP	cyclic pyranopterin monophosphate
nv	normal values
ACMG	American College of Medical Genetics and Genomics
